# Dyslexia—Early Identification and Prevention: Highlights from the Jyväskylä Longitudinal Study of Dyslexia

**DOI:** 10.1007/s40474-015-0067-1

**Published:** 2015-10-16

**Authors:** Heikki Lyytinen, Jane Erskine, Jarmo Hämäläinen, Minna Torppa, Miia Ronimus

**Affiliations:** Inclusive Literacy Learning for All, Agora Human Technology Center & Department of Psychology, University of Jyväskylä, P.O. Box 35, Jyväskylä, 40014 Finland; Agora Human Technology Center, University of Jyväskylä, P.O. Box 35, Jyväskylä, 40014 Finland; Department of Psychology, University of Jyväskylä, P.O. Box 35, Jyväskylä, 40014 Finland; Department of Teacher Education, University of Jyvaskyla, P.O. Box 35, Jyväskylä, 40014 Finland

**Keywords:** Dyslexia, Longitudinal, Prediction, Finnish, Intervention, GraphoGame

## Abstract

Over two decades of Finnish research, monitoring children born with risk for dyslexia has been carried out in the Jyväskylä Longitudinal Study of Dyslexia (JLD). Two hundred children, half at risk, have been assessed from birth to puberty on hundreds of measures. The aims were to identify measures of prediction of later reading difficulty and to instigate appropriate and earliest diagnosis and intervention. We can identify at-risk children from newborn electroencephalographic brain recordings (Guttorm et al., J Neural Transm 110:1059–1074, 2003). Predictors are also apparent from late-talking infants who have familial background of dyslexia (Lyytinen and Lyytinen, Appl Psycolinguistics 25:397–411, 2004). The earliest easy-to-use predictive measure to identify children who need help to avoid difficulties in learning to read is letter knowledge (Lyytinen et al., Merrill-Palmer Q 52:514–546, 2006). In response, a purpose-engineered computer game, GraphoGame^™^, provides an effective intervention tool (Lyytinen et al., Scand J Psychol 50:668–675, 2009). In doubling as a research instrument, GraphoGame provides bespoke intervention/reading instruction for typical/atypically developing children. Used extensively throughout Finland, GraphoGame is now crossing the developed and developing world to assist children, irrespective of the cause (environmental or genetic) of their failing to learn to read (Ojanen et al., Front Psychol 6(671):1–13, 2015).

## Introduction

From 1993, the Jyväskylä Longitudinal Study of Dyslexia (JLD) has followed, longitudinally from birth, a cohort of 200 Finnish children, half of whom are at familial risk for dyslexia. Dyslexia has many definitions and different criteria, but typically, the process of reading acquisition is unexpectedly impaired. From the antenatal clinic to the upper school years, the JLD children have been assessed on a plethora of neuropsychological, neurophysiological, cognitive, behavioral, and observational indices. Over time and in keeping with developments in technology, we can now present a retrospective confirmation of the risk factors and best predictors of developmental dyslexia. By comparing the JLD children’s early developmental measures with their current developmental status, it is possible for us now to demonstrate those indices that are the most salient predictors of later difficulty in reading skills. In turn, this allows us also to target a program of intervention toward these salient areas of difficulty at the earliest possible time, using our remediation-based technology, GraphoGame^™^ (GG) that has been developed alongside the JLD study.

### The Etiology of Dyslexia

Many children are denied the opportunity to become competent readers for a number of reasons. These reasons may be environmental, such as a lack of teachers/teaching facilities (see, for example, UNESCO EFA, 2014 [1]). For others, a biological basis (e.g., dyslexia) may be the underlying cause of a severe bottleneck to competence in literacy [2–5]. Repeated failure often results in apathy toward and consequent avoidance of reading-related activities.

Historically, the majority of developmental dyslexia research stems from either the UK or USA. Prevalence rates vary according to diagnostic criteria, although a generally accepted figure is less than 10 %. Etiologically, dyslexia is considered to have a genetic basis [6, 7], probably due to an interaction of several different genes. The first candidate gene was identified on the basis of the JLD data [8]. The hereditable ramifications of reading have also been well documented in twin studies [9] although the gene-environment interaction in combination with the unique heterogeneity of individuals themselves is a vital influence [10–13].

Hereditability has also been examined by following the development of children born to families with a dyslexic parent(s) (at-risk families) [14–17]. Family risk studies, such as the JLD, facilitate scrutiny of the gene-environment interaction in its natural setting. In family risk studies, the risk for dyslexia has been reported to range from fourfold to tenfold for children born with family risk depending on the applied criteria [18, 19]. Furthermore, the severity of the child’s reading difficulty is predicted by the severity of their dyslexic parent’s difficulty [20].

### The Impact of Orthographic Transparency

The nature and predictors of dyslexia differ, depending on the writing system or orthography. Among alphabetic orthographies, Finnish is one of the most transparent. Transparency of a writing system refers to the consistency of links between sounds or phonemes in speech and the graphemes (letters, letter clusters) that represent them in the text. The consistency at the grapheme-phoneme level is 100 % in both directions (e.g., in reading, the letter ‘a’ always represents the sound /A/, and in spelling, the sound /A/ is always represented by the letter ‘a’). The phonetically transparent structure of the Finnish language plays an important role in the acquisition of reading in the typically developing context.

Finnish children enter school in August of the year that they turn 7 years of age. By this time, 45 % can read [21] and the majority are at least familiar with most letter names. This happens before they experience any formal instruction in reading. Children are exposed to letters in Finnish kindergartens although the curriculum does not include formal reading instruction before school. After a few months in school, most children can decode words and also pseudowords, because the letter-by-letter decoding is not affected by the meaning of the word and differs from how decoding works, for example, in English. The situation for readers in Finnish stands in marked contrast to beginning readers of English as a most non-transparent alphabetic language. Children learning to read English require two more years of school instruction to reach the level of their Finnish counterparts [22]. It must be noted that the English-learning children begin formal instruction 2 years earlier and the learning burden per se of the more complex orthography must also be taken into account.

The disparity in learning burden as a function of the transparency of the language is marked. Finnish children must learn to master the sounds of fewer than 30 letters/graphemes, and these can be relied upon to be perfectly consistent in their sound/written representation. In contrast, the much heavier burden of English, with its many-on-one permutations on the journey from sound to speech and back to sound (and further exacerbated by inconsistencies between the reading-spelling and spelling-reading directions in the translation of sounds/letters), means that a child must master numerous context-dependent permutations from the outset. Consider, for example, the sounding of words such as ‘*key*’ and ‘*see*’ and contrast with ‘*quay*’ and ‘*sea*’. Indeed, the most consistent spelling to sound segments are typically larger than two letters in English [23]. Due to the lower burden in learning to decode accurately, dyslexia in transparent orthographies is typically characterized by difficulties in fluency of decoding, rather than simple accuracy [22, 24•, 25, 26].

English has many complexities, but one of the few complexities of Finnish is that an audible increase in the duration of the phoneme in the pronunciation is marked by repeated or double letters. In short, the manipulation of phonemic length or quantity. For example, in order to distinguish between the Finnish words ‘*mato*’ [worm] and ‘*matto*’ [carpet], the speaker is required to pause mid-phoneme in the latter example, and this signals to the listener that two letters ‘*t*’ are present. This feature has been recognized as one key area of difficulty to the Finnish dyslexic, particularly in spelling. The ability to perceive the lengthening of the phoneme in spoken Finnish is linked to the ability to spell the word accurately and is a major challenge for those with spelling difficulties in Finnish, as shown by results of the JLD. This Finnish complexity is a key feature accommodated in many of the experimental designs manipulated throughout the JLD studies (further detailed below).

Note that we focus here on findings among children learning to read in Finnish. In Finland, we have two official languages, Finnish and Swedish. Swedish is a Germanic language like English and not as transparent as Finnish. Swedish is mainly spoken in western coastal areas, and the vast majority of Finnish children are not bilingual. English lessons start in grade 3 and Swedish lessons in grade 7. The children of JLD are all Finnish-speaking, not bilingual children.

## The Jyväskylä Longitudinal Study of Dyslexia

Funded by the Academy of Finland and designated a Centre of Excellence for the initial 12 years, the JLD is a longitudinal study of 100 children at risk for developmental dyslexia (indexed by at least two first-degree relatives—such as mother and her close relative—with reading difficulties) and 100 age-matched peers with no known familial history of reading difficulties [27].

The earliest measures with the children concentrated on differences between at-risk and control groups. Within days of birth, brain event-related potentials (ERPs) in response to changes in vowel duration within consonant-vowel syllable sounds (such as /ka:/ vs. /ka/) were measured [28]. Group differences emerged in terms of hemispheric preference for right hemisphere processing in the risk group vs. left hemispheric preference for the non-risk group. Furthermore, more pronounced right hemisphere processing of consonant-vowel speech sounds (e.g., /ba/, /da/, /ga/) was also apparent in the newborn JLD risk children compared to control children [29].

Fast forward a decade and we can now confirm that the first indications of risk can be observed at a few days old. The brain ERPs measured at 3–5 days of age now demonstrate a significant predictive correlation to reading at second grade [30, 31•, 32]. Waveform differences seen in the ERPs as a function of group membership also show correlations with measures that reflect the earlier steps toward reading skill [33–35]. These group differences and correlations seem to persist to later preschool and school age, as revealed by ERP measures at 6.5 and 9 years [31•, 36–38].

By 6 months of age, toddlers in the risk group demonstrated difficulty with the discrimination of phonemic length at the behavioral level [39]. The risk children required a longer duration of this pause (40 msec more than their non-risk counterparts) to discriminate the difference between two pseudowords with short (/ata/) vs. long (/atta/) phonemic quantity. In addition, the ERP to changes in the same phoneme duration contrast within the auditory stream differentiated risk from non-risk [40].

Importantly, these measures now also predict letter knowledge and reading fluency [32]. Furthermore, as mentioned, problems in phonemic processing may have their origin in even more basic cognitive mechanisms. Those at-risk newborns who ended up facing reading difficulties at age 8 had atypical ERPs to sound frequency changes and showed compromised perceptual differentiation of phonemic duration, as well as a correlation between newborn ERPs and school-age phoneme duration discrimination [41]. Problems in phonemic length discrimination seem to be persistent and observable still within the first three grades in school [42, 43•].

By the time of emergent speech, differential development of spoken language skills is the earliest behaviorally observable indication that has predictive relations to the acquisition of written language skills. A small (~15 %) portion of children start speaking later than expected. This late-talking phenomenon can have three forms: delayed receptive (comprehension of spoken language), delayed expressive (articulated language), and delay in both receptive and expressive language [44, 45]. Most notably, children with a double impairment in both receptive and expressive language were shown to be at much higher risk of developing later reading and spelling difficulties, but only those in the risk group. Comparable relationships were much weaker in the control group [45]. The inclusion of family risk is apparently a critical factor in the relationship between early language difficulties and subsequent prediction of difficulties with literacy at a later age [46].

Finnish letter names are near synonymous with letter sounds, and awareness of Finnish phonology is considered to be near synonymous with awareness of letter sounds. The best early predictors of dyslexia in addition to familial incidence of dyslexia are a child’s phonological awareness, letter knowledge, and rapid naming [18]. These three main predictors of dyslexia have been replicated in many other studies in different orthographies [18, 47–51]. What is striking about these skills is that they are strong predictors of age 8 years (grade 2 spring) reading, already years before school entry (i.e., at age 3.5 years) [18, 52, 53]. Even though it is possible to demonstrate valid prediction of later reading difficulties from age 3.5 years on measures of rapid automatized naming (RAN) and phonological awareness, probably, the most parent-friendly way to identify children who are in need of help is the follow-up of their readiness to store letter sounds, as many children who were late in learning to read at the end of the second grade were comparably late in their letter knowledge years before school entry. It is, however, important to take into account that this is applicable in an environment where children have an opportunity to be exposed to letters, as is the case in Finland, where letters are on the walls of the preschool environments. Consequently, this measure is at risk of showing false positives. However, if a child has spontaneously stored >10 letter names before school entry, it is safe to expect that he/she will not have severe problems in learning to read.

In transparent orthographies, the impact of letter name learning which supports directly the development of phonemic processing is particularly easy to understand, as the initial focus of learning to read is to build connections between the sounds of single phonemes and their representative letters/graphemes. Therefore, any difficulty with the differentiation of the small speech units (phonemes) or letters may manifest as a substantial bottleneck. However, after basic decoding skill is acquired, decoding should automatize to become fluent. This means that efforts should be made to ensure that children read material which motivates them to apply their new skill as much as possible.

The prediction of fluency development has been demonstrated in various orthographies through RAN [54]. More recently, we have shown that the relative importance of phonological awareness, letter knowledge, and rapid naming varies according to grade level and the reading skill in question [55•]. Predominantly, phonological awareness predicts reading accuracy and rapid naming predicts reading fluency [21, 56]. Consequently, the impact of phonological awareness is limited to the early phases of reading acquisition while the impact of rapid naming is higher when fluency is increasingly the skill in focus. The predictive strength of phonological awareness diminishes earlier in a transparent as opposed to non-transparent orthography because the learning burden placed on phonological processing is low—letters help children living in transparent writing environments to store the phonemes quickly in long-term memory—and reading accuracy soon hits ceiling [57].

Although the strongest predictors of reading development are phonological awareness, letter knowledge, and RAN, note that the children with dyslexia in grade 2 also had lower early performance in vocabulary, verbal short-term memory, and morphological skills at age 2 years onward in comparison to children who did not develop reading problems at school age. Table 1 shows the significant predictors of compromised reading acquisition by second grade when the majority of Finnish children are accurate readers. The most significant predictors, spanning 2 years of age to school entry age, are listed. Those measures where groups with and without familial risk differed significantly as a function of confirmed dyslexia diagnosis are also shown. Our findings also show how cognitive skills develop interactively from early on and also other skills predict the development of phonological awareness, rapid naming, and letter knowledge [58]. In addition, early cognitive development is also heterogeneous and dyslexia can result from different profiles [24•, 59].

**Table 1:** Significant predictors of second-grade reading skill for the JLD children

Predictive correlates to second-grade reading skill and group differences in relation to confirmed dyslexia for the JLD children						
Age (years)	Measure	Prediction of reading skill (2.gr)	Group differences; dyslexia vs no dyslexia among all JLD children		Group differences; family risk with dyslexia vs. family risk with no dyslexia	
		*r*	*F*	Effect size	*F*	Effect size
2	Maximum sentence length	0.204**	8.41**	0.49	5.38*	0.47
2	Articulation accuracy	0.110	7.34**	0.45	9.93**	0.64
3–5	Inflectional skills	0.249***	11.84***	0.56	6.33*	0.51
3–6	Phonological sensitivity	0.479***	28.93***	0.94	8.76**	0.63
4–6	Phonological manipulation	0.418***	24.87***	0.91	11.48***	0.73
5–6	Verbal short-term memory	0.344***	12.55***	0.57	13.63***	0.75
5–6	Letter knowledge	0.547***	50.93***	1.26	18.52***	0.92
5–6	Naming speed	0.501***	51.79***	1.06	19.22***	0.85
School entry	Reading accuracy	0.653***	77.52***	1.06	32.88***	1.01

Effect sizes for group differences for later diagnosis of dyslexia amongst all JLD participants, as well as differences between the children with and without family risk are also shown

*p < 0.05, **p < 0.01, and ***p < 0.001

We [59] modeled, using latent profile analysis, the relationship between measures taken from all preschool ages from seven language-based skill domains—receptive and expressive language, morphology, memory, phonological awareness, letter knowledge, and naming speed using the entire battery of language measures obtained by grade 2. The aim was to examine the heterogeneous paths to reading among JLD participants. We differentiated four groups or pathways to reading: *declining*, *typical*, *dysfluent*, and *unexpected*. In the declining group, during the preschool years, phonological development was delayed amongst 35/199 of the children and their reading level by the end of the second grade was 1 standard deviation (Sd) from the norm of the other children. Those members of the dysfluent group (*n* = 12) indexed delayed early language and special difficulty in rapid naming of familiar objects at age 5.5. Members of the *unexpected* group had previously demonstrated good early language development, yet the mean level of reading achieved −0.5 Sd from the control mean during the second grade.

What was apparent here was that group membership was not exclusive as a function of risk. Neither were the demarcating characteristics of any group homogeneous. What did, however, emerge in a later analysis was that, although 50 % of the risk group indicated some level of reading-related difficulty, 33 % satisfied the criteria for dyslexia diagnosis by the end of second grade. In contrast, only 9 % of non-risk children met the criteria (for recent discussion, see Lyytinen 2014) [60•].

Of the home literacy environment (HLE) measures assessed before school entry, parent-child shared book reading has gained the most interest. Its overall effect on language and literacy development has been found to be less than 10 % [61–63]. The previous findings [61–65] indicate that evidence for positive effects of shared reading is clearest for oral language skills. For learning of letters, however, direct teaching of letter symbols is generally required [62, 64]. The more indirect measures of HLE, such as the number of books in the home and the parents’ literacy-related behaviors, tend to have a smaller impact on the child’s literacy and language development than parent-child shared reading experiences [61, 63]. With the exception of letter knowledge, the other predictors of decoding, such as RAN, memory, and phonological awareness, appear not to be highly associated with variation in HLE [62, 63].

Probably the easiest, most readily available and literacy-relevant measure for identification of children who need help to avoid problems in reading acquisition is letter knowledge. This is especially the case if the child has had a good opportunity to see letters. A ‘dynamic assessment’ pilot could be carried out to observe how easily the child stores the letter names. If this pilot is carried out close to the age of school entry and difficulty is apparent, it is better to move to letter sounds using a similar type of training delivered by the GraphoGame, which we recommend later in this paper.

In the fully transparent writing environment, the relationship between letter knowledge and phonemic awareness necessary for learning to read is so inter-twined that in modeling the prediction with letter knowledge (LK) measures taken at an earlier age than those of phonological awareness (PA), no common variance is left (from LK) between PA and reading accuracy which, however, is still partly predicted also by rapid naming. Phonemic awareness in transparent orthographies is most likely assisted by the visibility of the letters themselves. The child’s interest in naming these letters drives them to store the crucial information associated with their sounds. The realization of these letter-sound connections only serves to drive the child forward on the road to basic reading skill. Because the Finnish language is so transparent, letter sound knowledge and phonemic awareness are near synonymous, and consequently, once mastery of the alphabetic principle, i.e., sounds of the letters, has been achieved, reading is underway.

## GraphoGame

We have demonstrated that there are many strong and valid predictors of a child’s risk for later difficulties with reading. Not least, these include newborn measurements from the scalp surface, late talking and delayed rapid naming, letter knowledge, and PA. From a purely practical perspective (certainly from that of teachers’ and parents’), probably, the most easy-to-administer predictor of later reading difficulty in risk children is delayed letter knowledge. This latter measurement also complements well the structure and function of the GraphoGame platform that is primarily designed to address these difficulties.

**Fig. 1:**
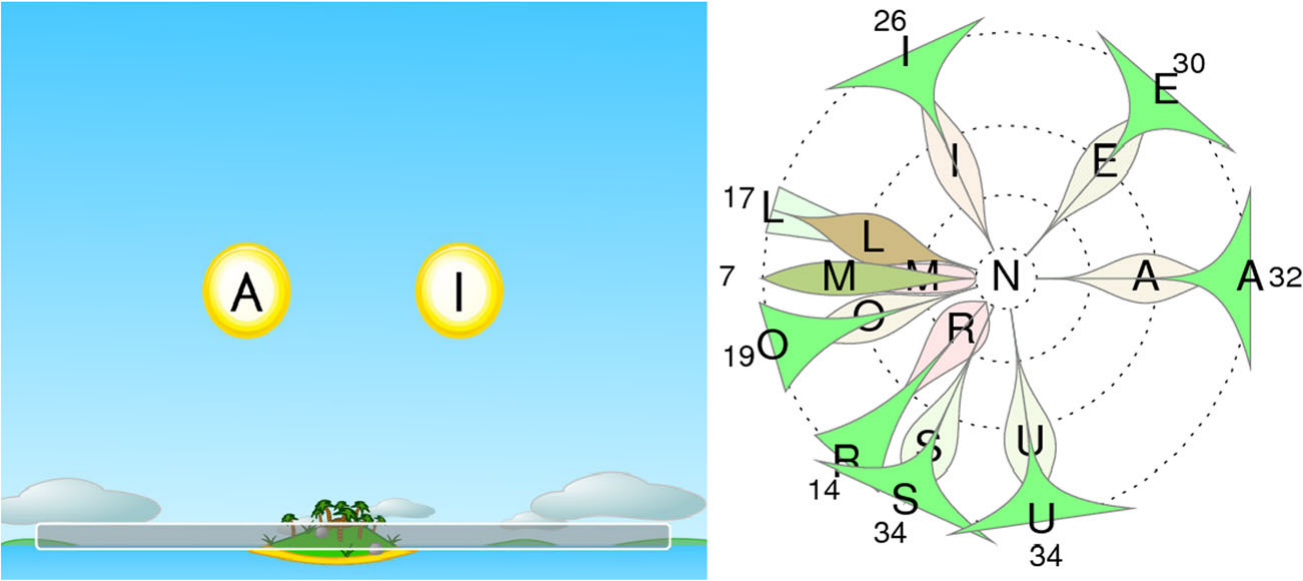
In the game (left-hand-side), the learner chooses, from the alternatives on the screen, the letter that corresponds to the sound heard through headphones. The illustration (right-hand-side) shows how the game data can be analyzed. The illustration shows the sound /N/ (in the center) which the learner has heard in the game more than 100 times by the time of this analysis, and the incorrect alternative letters (distractors) shown on the game screen at the same time as letter *N*. The *numbers on the outer circle* tell the number of times that a certain distractor was present on the game screen at the same time with target letter *N*. From the illustration, it can be seen that, in the beginning, the learner has chosen letter *M* or letter *R* when *N* was asked for (shown in *red*) but has later learned to differentiate *N* from these letters (shown in *green*). Similar improvement can be seen in other letters (for more information about this method, see Lyytinen et al. ) [52]

The training game GraphoGame (GG) (in Finnish, ‘Ekapeli’) is a digital learning environment that we have developed to support at-risk children’s reading acquisition (see www.lukimat.fi). GG—an extension of the Finnish Ekapeli in order to make the same concept applicable to learning the writings of other languages also—began as an EU-Funded Marie Curie Excellence Grant awarded to Prof. Ulla Richardson. The Finnish Ekapeli has been under development since 2003. From 2008 to the time of writing this, it has been funded by the Ministry of Education and Culture for use, without charge, to all Finnish children. The plan is to have GG developed to the same extent for use also in other countries. The learning game, also comprising training of basic math skills, is used in schools and homes across Finland on a daily basis by thousands of children. Since the service was launched in 2008, more than 270,000 children in Finland have played GG, ranging from kindergartners practicing letter-sound connections, to older children training reading fluency. These are respectable numbers because one age cohort in Finland has around 60,000 children. Besides being an educational tool, GG is also a research instrument as it saves the player logs, which can be used to analyze the learning processes of the players, including the identification of specific areas of difficulty (see Fig. 1 (right-hand)).

Figure 1 (left-hand) shows an extract from a GG learning task for a transparent orthography where single letters are shown on the screen. In the game, the player hears a sound and matches it with the appropriate letter. A balance between repeated items and the introduction of novel items ensures sufficient exposure to facilitate storage to memory. The unique adaptive nature of the software ensures that the content of each trial is determined by the player’s performance in the previous trials, providing an optimal level of challenge for each individual player. In the initial stage, with few alternatives, the Finnish player learns to connect single easy-to-differentiate sounds to single letters. Play then progresses to more difficult-to-differentiate sounds, such as those of /N/ and /M/ with a larger number of alternatives. In keeping with the player’s development, play moves then to larger units, i.e., to syllables, words, and pseudowords. The proceedings are slightly different in other languages and substantially different in GG which is implemented for learning to read non-transparent writing, such as English [66]. In accordance with ‘grain size theory’[23], beginning readers require to isolate consistencies or ‘shared grain sizes’ in the language so that they can be reliably generalized to the reading of novel words. In transparent languages, these ‘grains’ are small (one letter has one sound). In contrast, ‘small grains’ in English are inconsistent, but larger grains, such as rimes, are more consistent. The connection building should therefore be focused on the more consistent units, which means that the English units should be larger in size from the outset.

After basic decoding skill is achieved, the training can continue with the fluency version of GG, which focuses on improving children’s reading speed by providing training in syllable recognition, reading of sentences, and longer texts. The different versions of GG sustain children’s motivation to play, for example, by child-friendly fantasy contexts, reward systems, and the introduction of new types of learning tasks as the player progresses in the game. A study has shown that the motivational features of GG seem to increase children’s interest to play, especially at the beginning of the training [67•].

It is recommended that the children who are at risk of reading difficulty and who ultimately will require greater practice than their peers, start to train with GG just before the beginning of formal schooling. The aims are to negate any overt difference between the child and his/her peers by the time that school begins and to avoid the potential negative motivational consequences that may emerge when the child sees him/herself as a slower learner [68].

GG’s capacity to support special teaching [69] is demonstrated in that struggling first graders who were allowed to use GG as part of the remediation hours were able to catch up with their peers by third grade while those who participated in face-to-face remediation only, failed to do so. GG has also been shown to support the development of reading fluency [70, 71•]. Further information is available from info.graphogame.com.

Reading-related research involving GG and its implementation is now underway in more than 30 countries with those children in most need. For example, African children receive the most urgent attention from the perspective of the Finnish developers and their African colleagues. For interested parties, the work by Ojanen and colleagues [72, 73•] outlines the latest developments and info.graphogame.com provides regular updates.

## Conclusion

The typical reader of transparent writing (e.g., Finnish, German, Italian, and Spanish) is able to quickly grasp the simple relationship between letters and sounds, assemble, and manipulate these to form the words of the language and progress, with prolific reading and literacy experience, toward fluent reading with comprehension. In contrast, readers with risk for developmental dyslexia, despite the simple nature of the language, may encounter bottlenecks to their proficiency. Such bottlenecks may have a knock-on effect in the progression toward the achievement of full literacy. Difficulty with discrimination of complexity, such as phonemic length in Finnish, may hinder competence in letter-sound acquisition. In turn, overly focusing on these letter-sound elements may handicap progression toward fluent reading. Reading that lacks fluency is disrupted in nature and can never therefore become fully automatic. Unless automaticity is achieved, comprehension can never be fully comprehensive due to working memory limitations. It is important therefore for intervention to interrupt this potential impasse by focusing the learner’s attention quickly away from letter-sound drilling toward larger units (as is the case with non-transparent languages where letter-sounds are often learned concurrently with larger, often exceptionally spelled but high-frequency units).

Now that Jyväskylä’s children, who were born with familial risk for dyslexia, are older, we are able to confirm the presence, extent, or absence of their reading difficulty. Furthermore, because we have deployed a plethora of measures over a continuous period from birth, not only can we confirm those measures that differentiate children with difficulty from those without difficulty, but we can also confirm the measures that act as predictors of such difficulty later on. The negative consequences of growing up to be different from one’s peers cannot be underestimated at the level of the classroom, in terms of not only peers’ noticing and acting upon such differences, but also the impact on the self-esteem and confidence of the individual in difficulty. It is therefore crucial, at the earliest possible opportunity, not only to identify this potential for difficulty, but also to intervene in order to minimize the appearance of difference per se, for the vulnerable child.

Our longitudinal research has demonstrated that there are numerous measures that can predict a child’s potential for later difficulty with literacy. From a practical perspective, however, and one not requiring complex and expensive technology, the most salient indicators of later difficulty lie with expressive language delay and delay in acquisition of the names of letters. It must be acknowledged that there are a plethora of speech and language measures (e.g., PA, rapid naming, family literacy environment) that exert an influence on a child’s later difficulty. However, what we are suggesting is that close attention should be paid to those children who display delayed language and/or who may not be grasping the letters of the alphabet in line with expected developmental milestones. A program of intervention, such as GG, can then be implemented. In the earlier years, content could involve introduction to more meaningful larger units in whole word form. This would help stimulate awareness of orthography and accumulation of vocabulary. Once sufficient cognitive maturity is reached at school entry, learners may progress to manipulation of the smaller most consistent units dependent on the orthography in order to foster the precursors of reading acquisition.
